# Supplementary material for: Impact of domain knowledge on blinded predictions of binding energies by alchemical free energy calculations
Source: J Comput Aided Mol Des. 2017 Nov 13;32(1):199–210. doi: 10.1007/s10822-017-0083-9 (PMC5767197; doi:10.1007/s10822-017-0083-9)
Supplement: Supplementary file 1 — Supplementary material 1 (DOCX 913 kb) [file 10822_2017_83_MOESM1_ESM.pdf]

Supplementary Information

Impact of domain knowledge on predictions of binding energies by alchemical free energy calculations

A. S.J.S. Mey, J. Juárez-Jiménez, J. Michel

September 29, 2017

Contents

1 Datasets **2**

2 Details of Alchemical free energy protocols for Set1 and Set2 **2**

3 Perturbation networks **4**

4 Scaling for LitSet1 and LitSet2 **8**

5 Comparison to other Protocols **10**

6 Complete ligand library **11**

1 Datasets

The chemical structure of compounds used for relative free energy calculations can be found in figure 1 and figure 2. The four different data sets, LitSet 1, LitSet 2, and D3R grand challenge set 1 and 2 are shown.

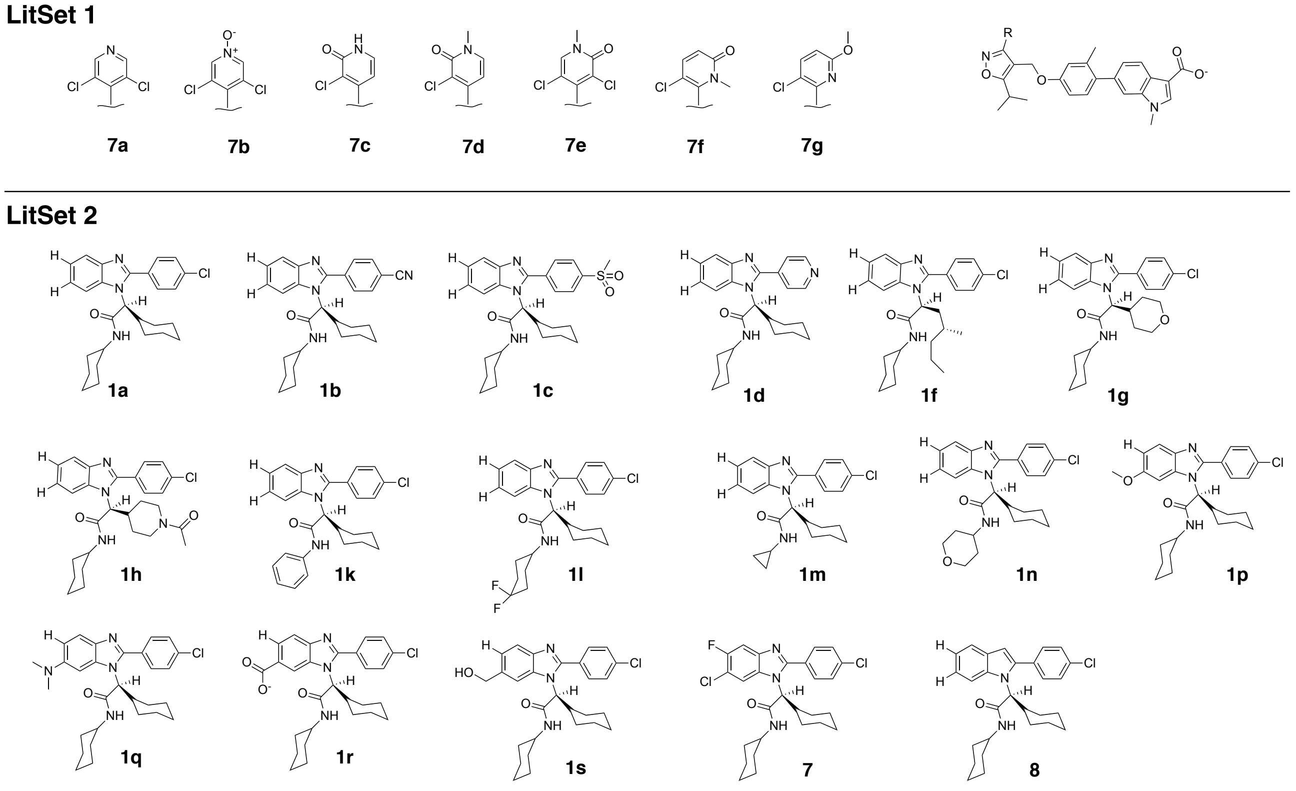

Figure 1: Compounds of the literature data sets used for the computations

Set 1

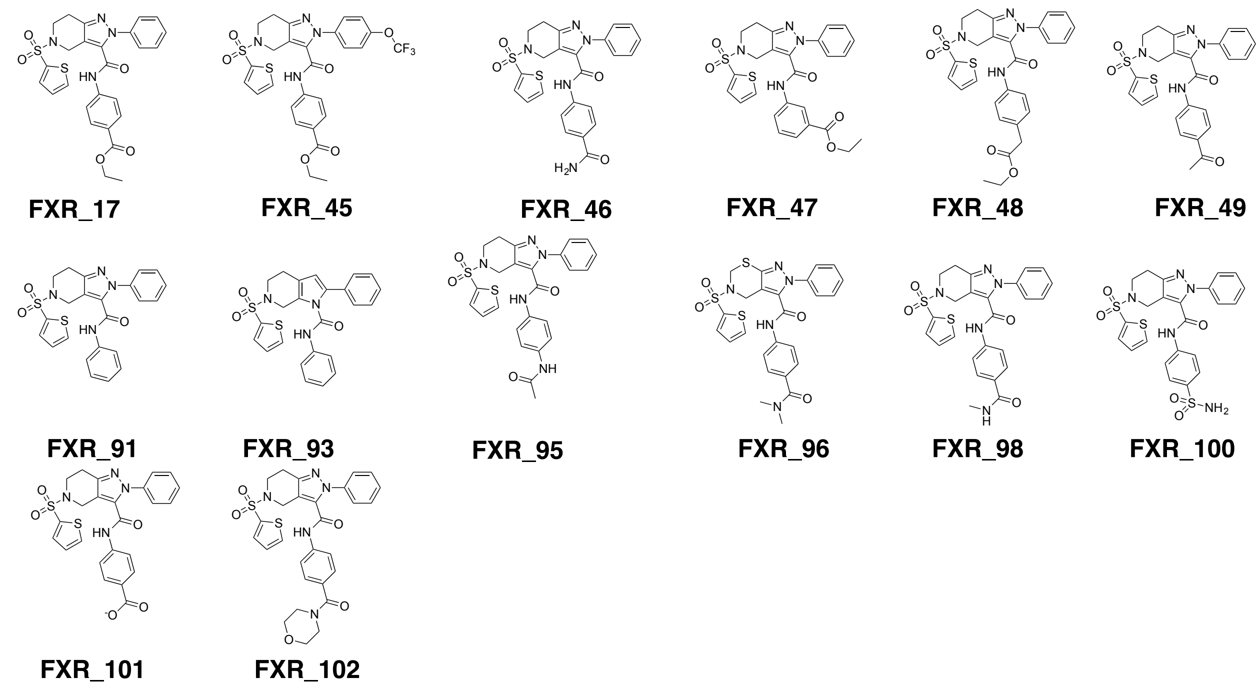

Set 2

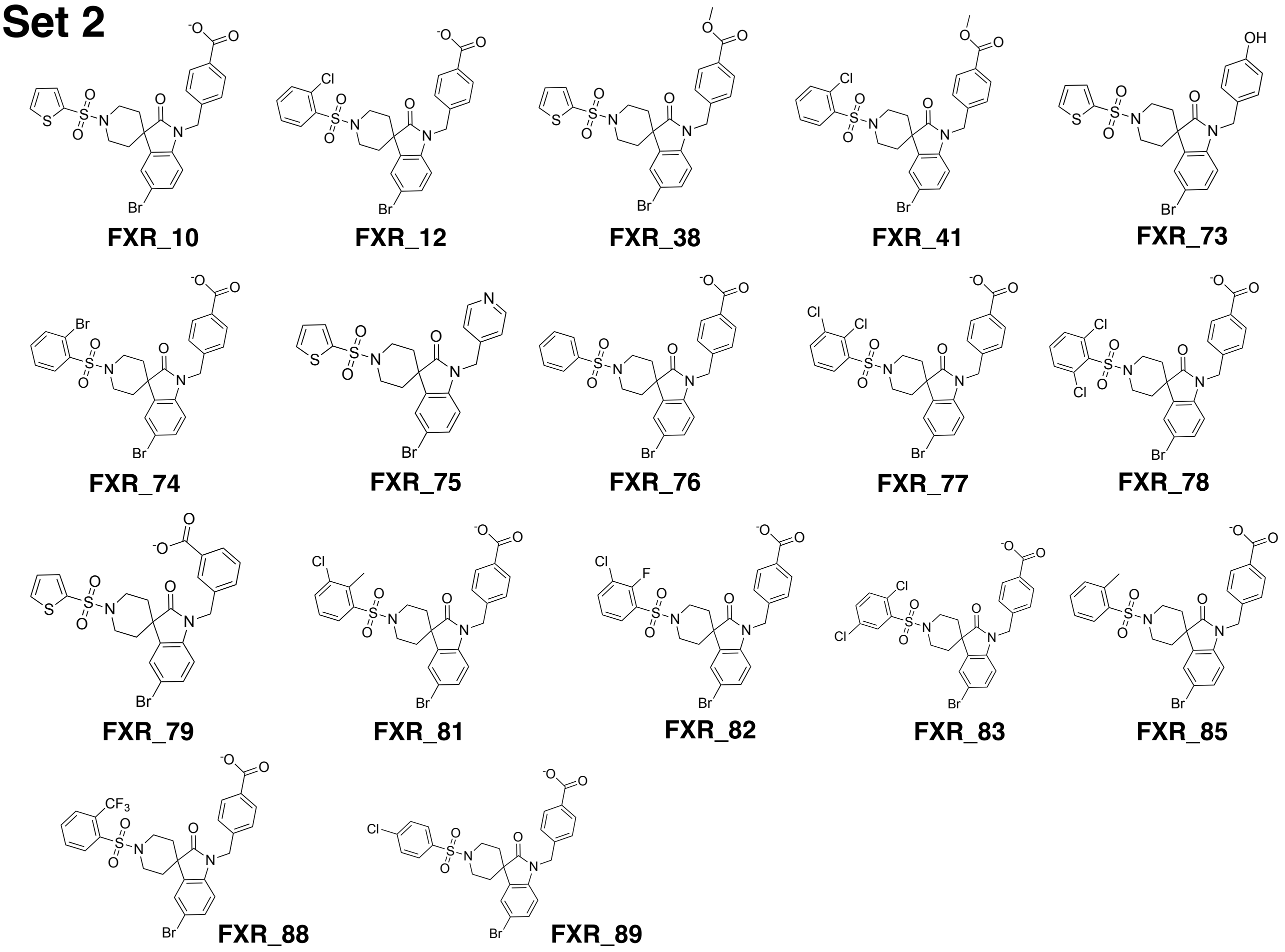

Figure 2: D3R grand challenge datasets set1 and set2

Table 1: Summary of perturbation λ windows for run1 and run2 of Set1 stage2, if perturbation not listed, then 17 windows were used.

Number of λ windows	
9	26
FXR99∼FXR91	FXR95∼FXR48
FXR49∼FXR46	FXR101∼FXR91
FXR46∼FXR49	FXR91∼INT02_BM2
FXR98∼FXR46	FXR96∼FXR91
FXR49∼INT01	INT01∼FXR102
INT01∼FXR49	FXR102∼INT01
	FXR91∼FXR96
	INT02_BM2∼FXR91

2 Details of Alchemical free energy protocols for Set1 and Set2

For set1 all perturbations were run twice. The following perturbations were additionally run, using the same number of windows as before and using a forwards and backwards perturbation: **49-91**, **102-INT01**, **INT02_BM2, 91**, **96-98** and **100-98**. For set2 data 68 of the perturbations were run using 9 windows, 22 using 17 windows and 18 using 26 windows and a summary can be found in file: jobtype_set2_stage2.dat, all perturbations were run twice and perturbation with irregularities were run 3 times. These additional runs were, both in forwards and backwards direction: **10_BM1-Int01_BM1**, **76-77_BM2**, **83_BM2,82_BM2** and **79_BM1B-Int01_BM1**. All relevant input files for repeating calculations and analysis can be found on github: `https://github.com/michellab/D3R_GC2_Data`.

3 Perturbation networks

The perturbation network of LitSet1 and LitSet2 can be found in figure 3 and figure 4.

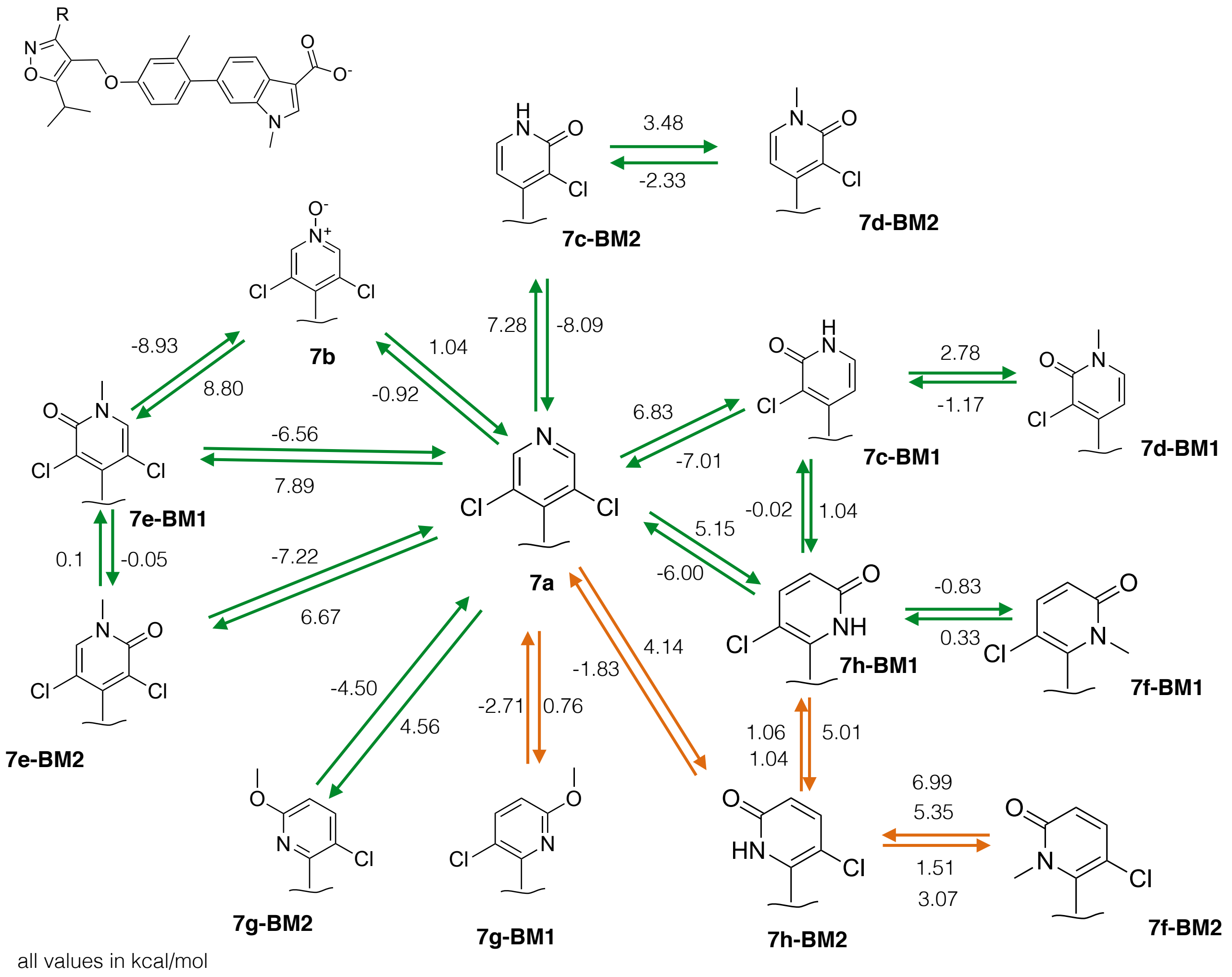

Figure 3: Perturbation map for LitSet1

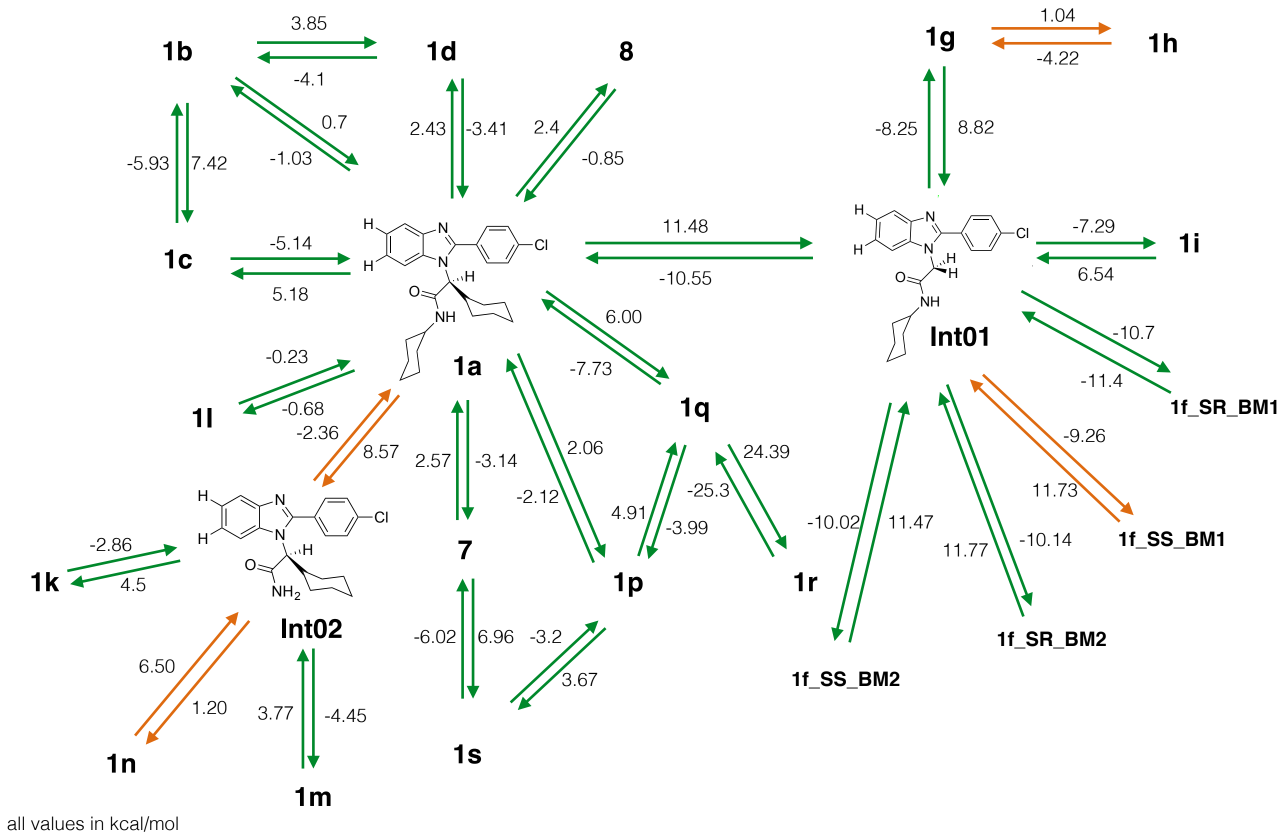

Figure 4: Perturbation map for LitSet2

The perturbation network of set1 and set2 of the D3R grand challenge 2 can be found in figure 5, figure 6, and figure 6.

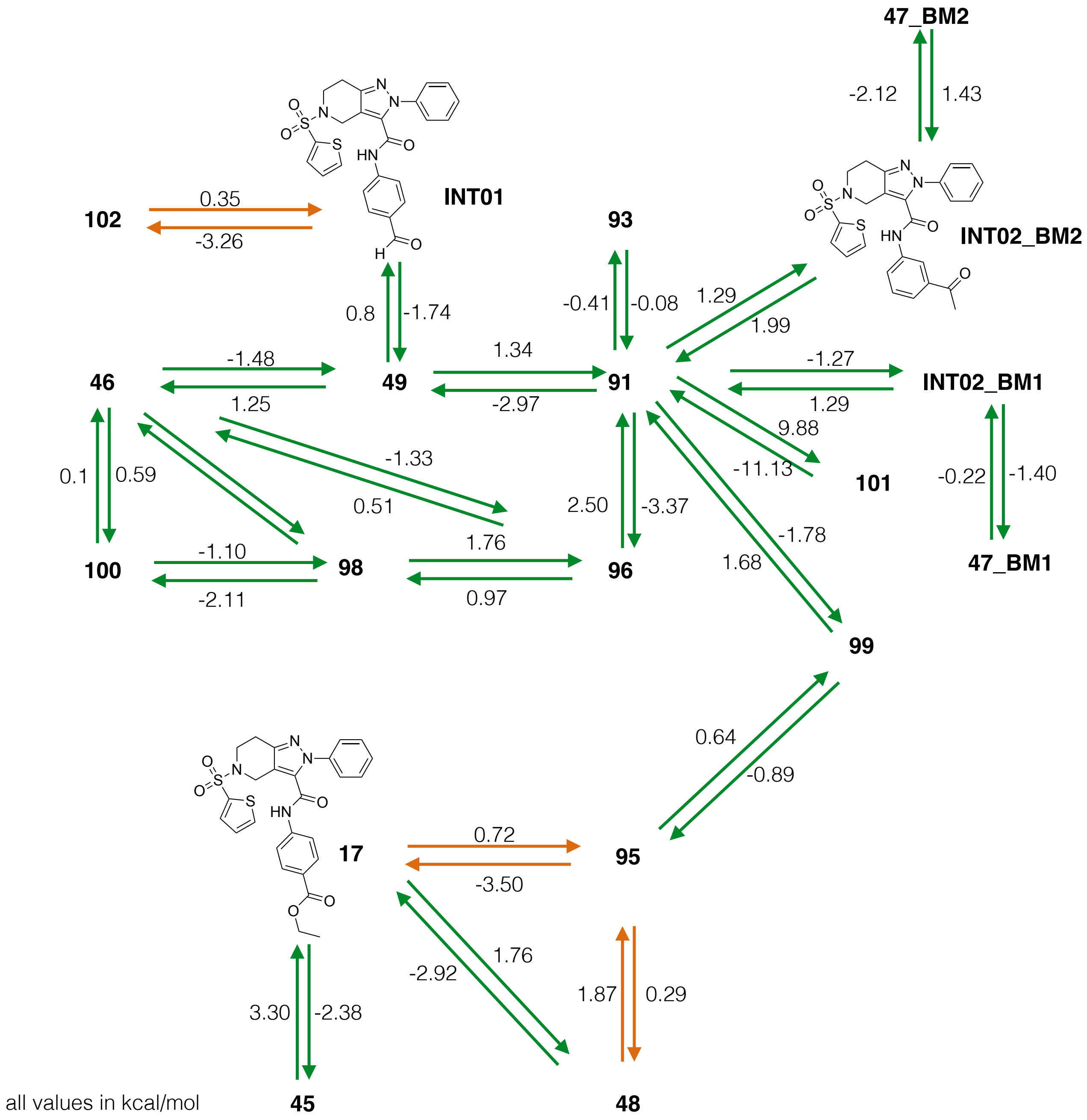

Figure 5: Perturbation map for set1 of the D3R grand challenge

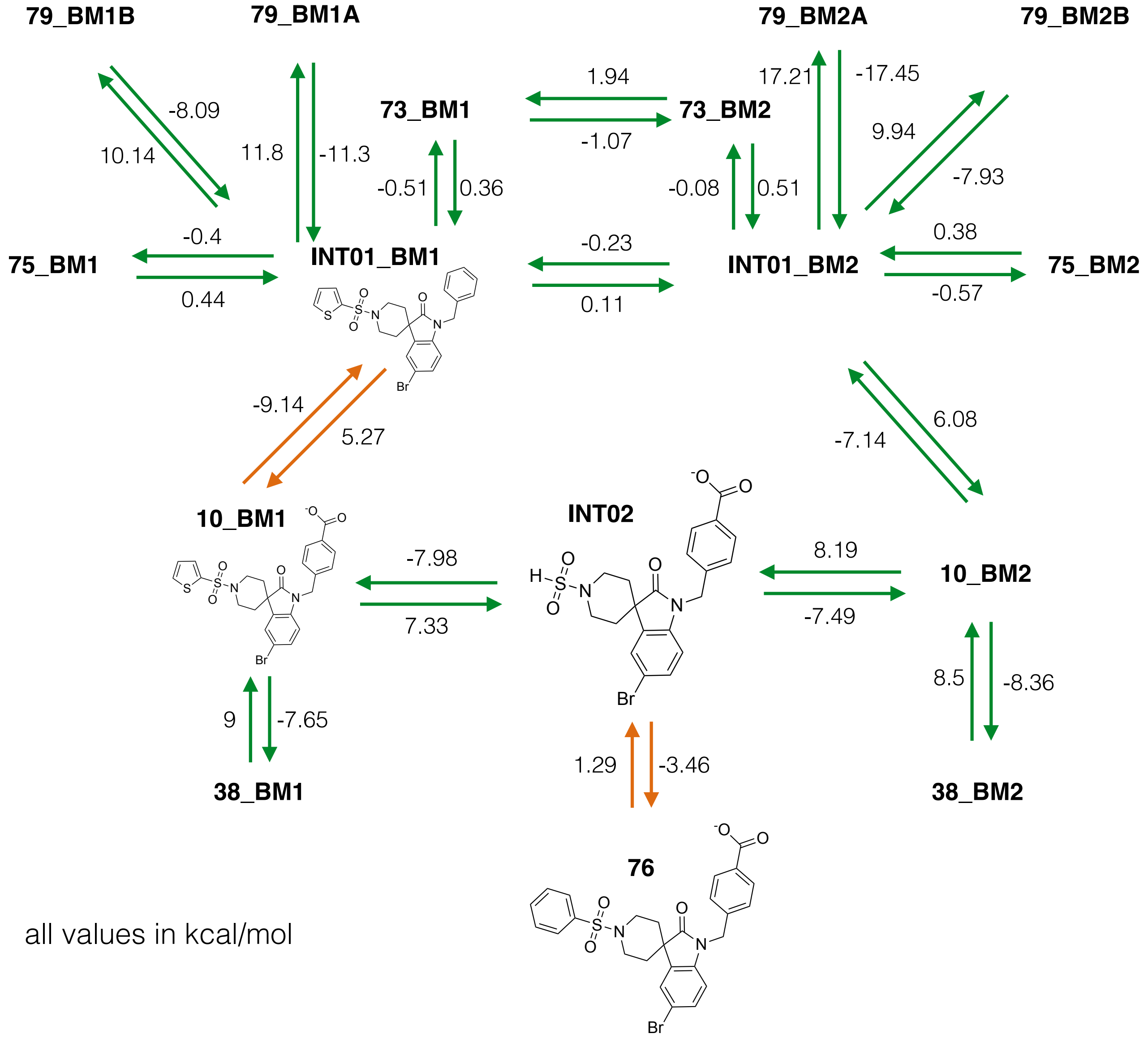

Figure 6: First part of the perturbation network for set2 of the D3R grand challenge

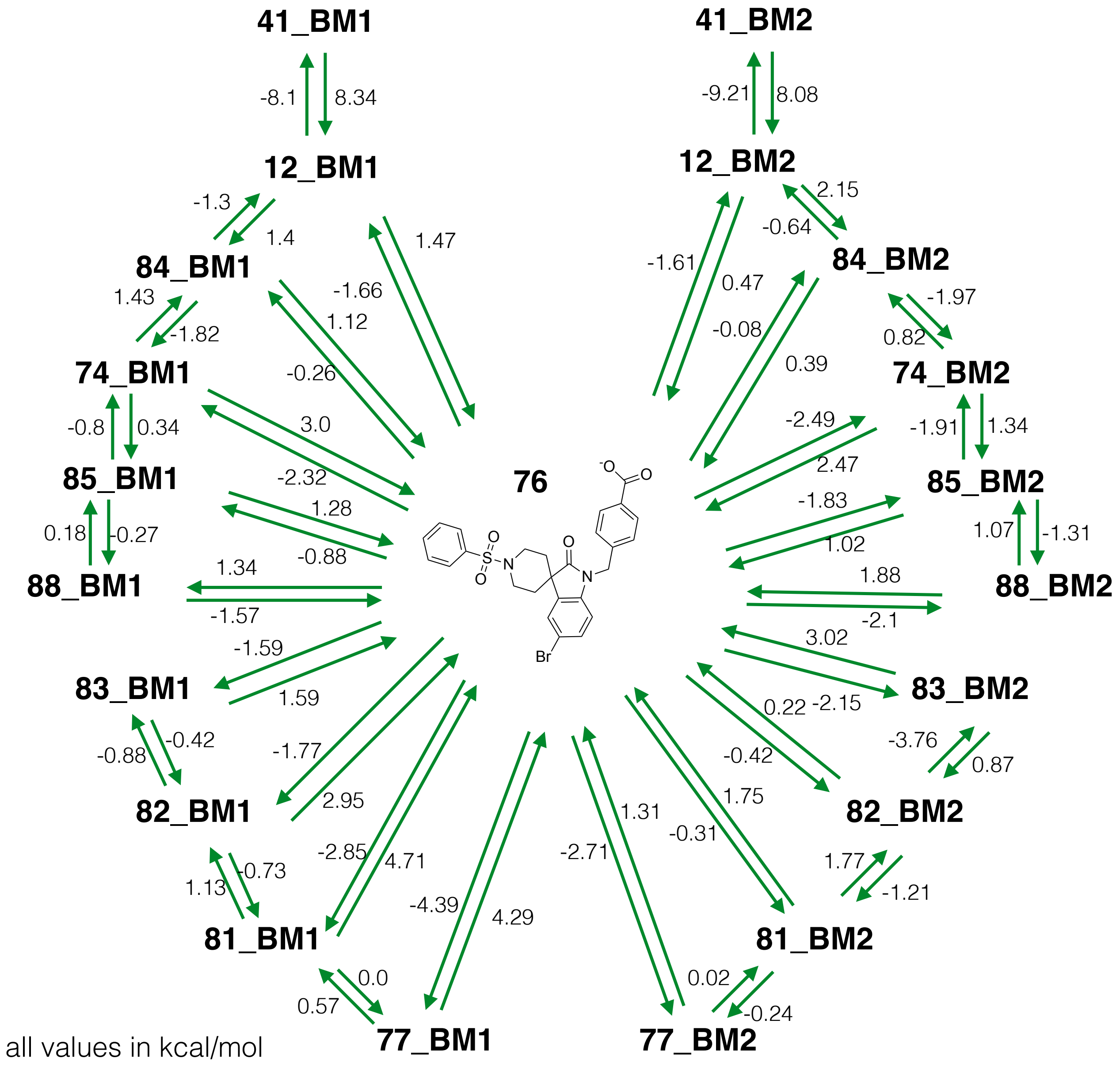

Figure 7: Second part of the perturbation network for set2 of the D3R grand challenge

4 Scaling for LitSet1 and LitSet2

Figure 3 and figure 4, show the scaling (1 - 0.5 and experimental values) of the estimated free energies (A) and the resulting Pearson correlation coefficient (B) for LitSet1 and LitSet2 respectively.

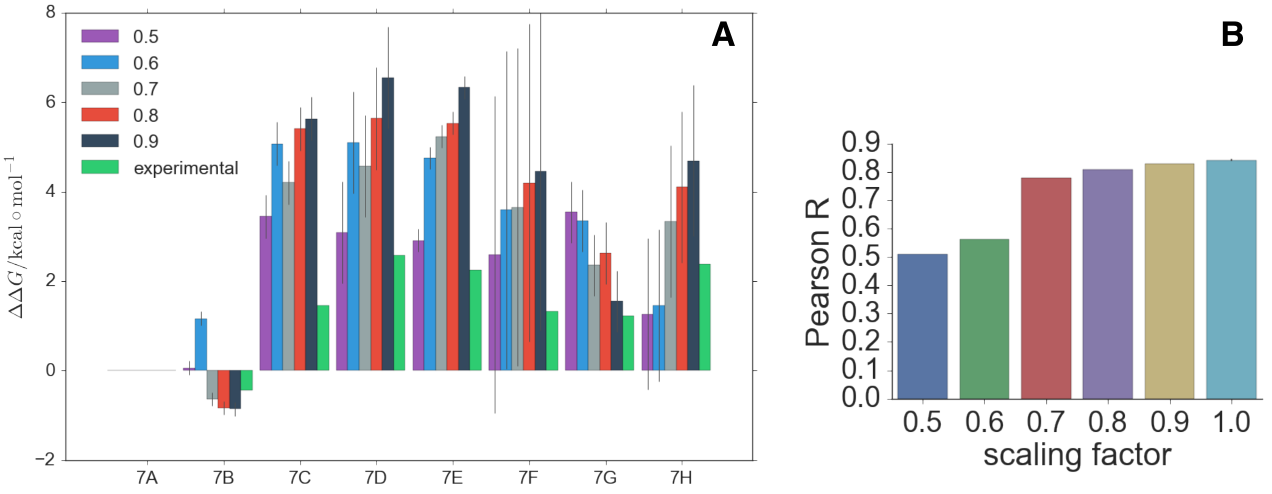

Figure 8: A: Scaling of estimated free energies for LitSet1 compounds, B: resulting correlation for scaling

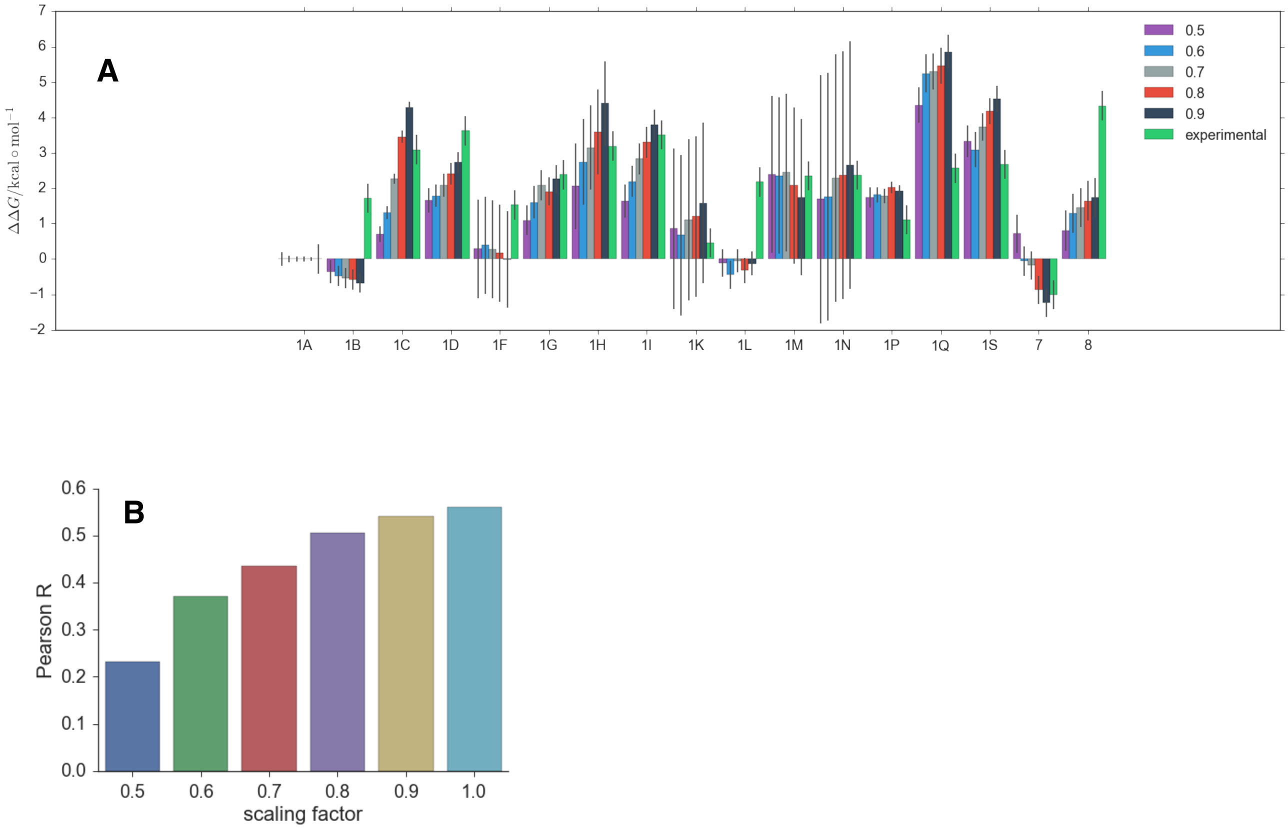

Figure 9: A: Scaling of estimated free energies for LitSet2 compounds, B: resulting correlation for scaling

5 Comparison to other Protocols

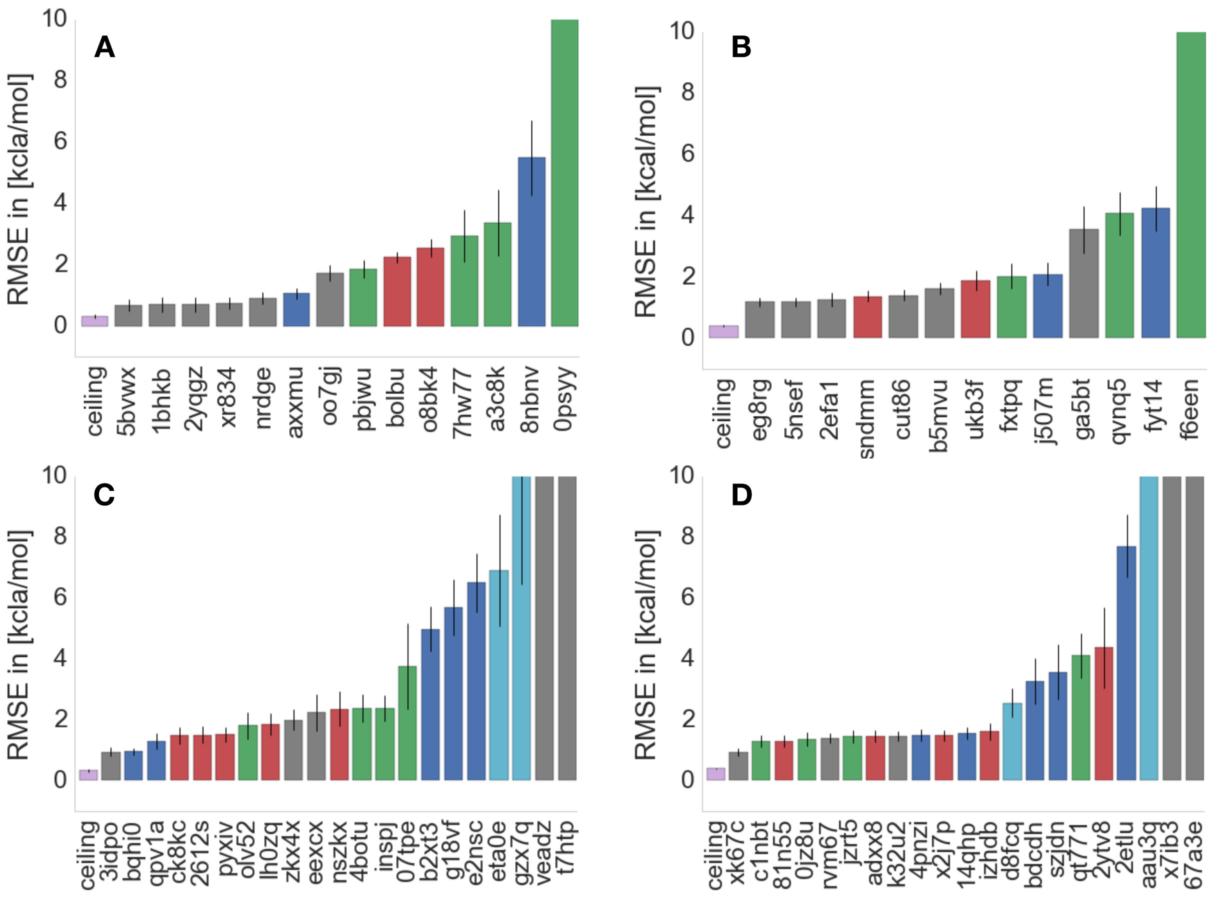

Figure 10: A: set1 stage 1, B: set2 stage 1, C: set1 stage 2, D: set2 stage 2. RMSD deviation of all submission protocols.

6 Complete ligand library

Figure 6 shows all 2D structures of the 102 inhibitors in the dataset provided.

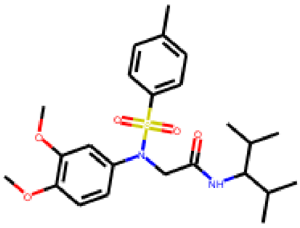

FXR_1

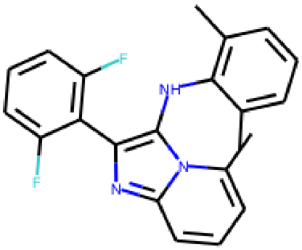

FXR_2

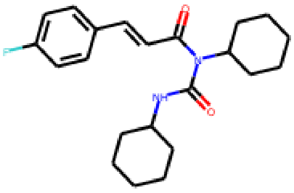

FXR_3

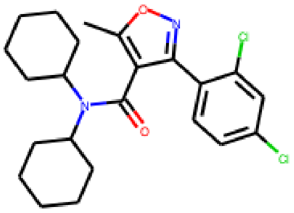

FXR_4

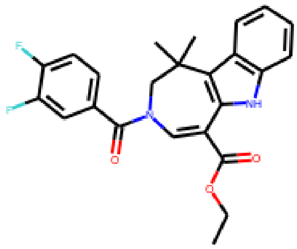

FXR_5

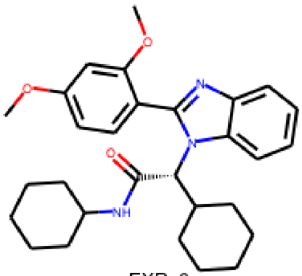

FXR_6

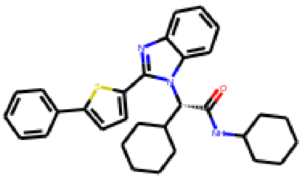

FXR_7

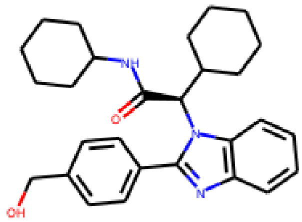

FXR_8

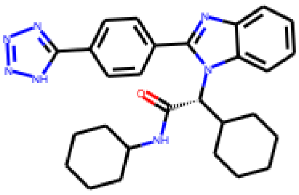

FXR_9

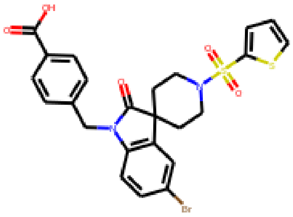

FXR_10

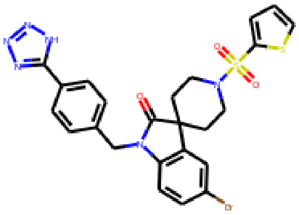

FXR_11

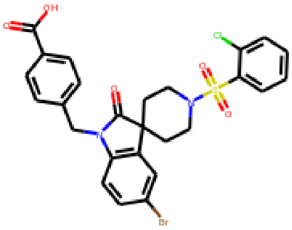

FXR_12

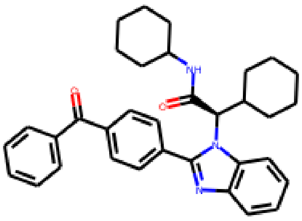

FXR_13

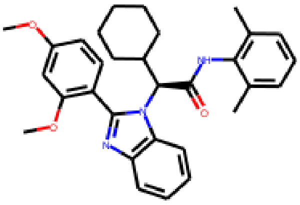

FXR_14

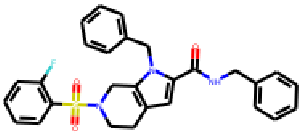

FXR_15

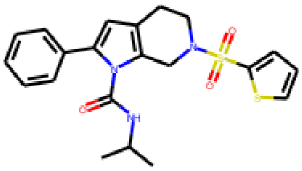

FXR_16

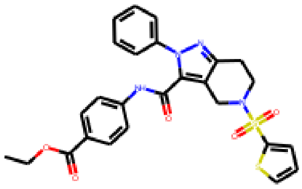

FXR_17

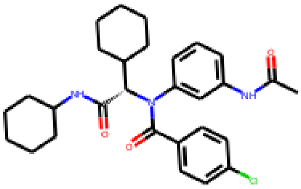

FXR_18

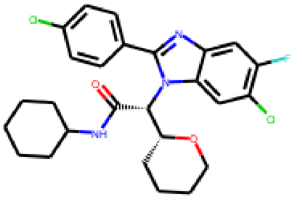

FXR_19

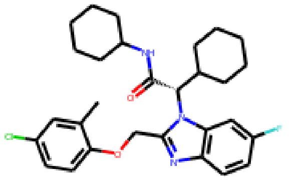

FXR_20

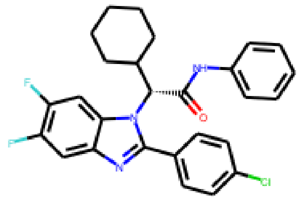

FXR_21

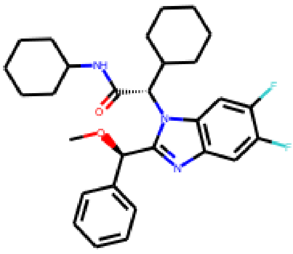

FXR_22

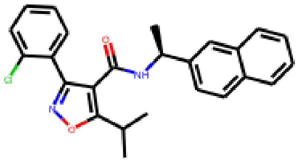

FXR_23

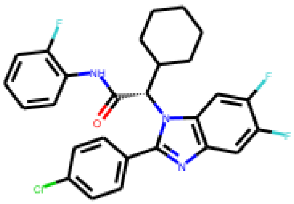

FXR_24

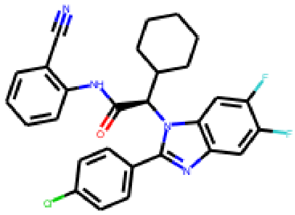

FXR_25

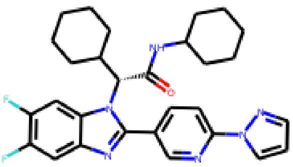

FXR_26

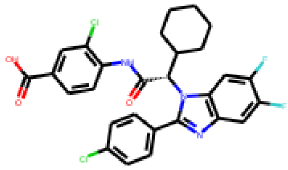

FXR_27

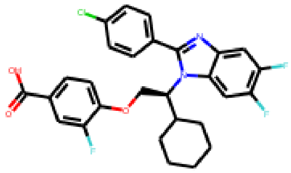

FXR_28

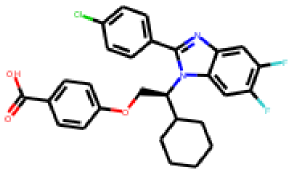

FXR_29

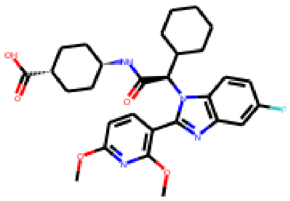

FXR_30

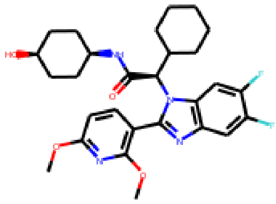

FXR_31

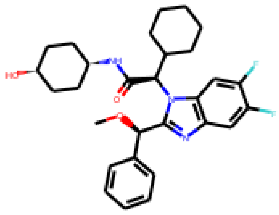

FXR_32

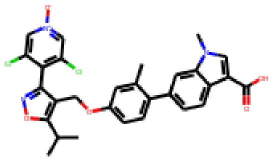

FXR_33

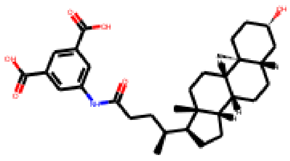

FXR_34

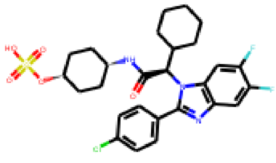

FXR_35

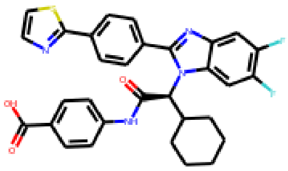

FXR_36

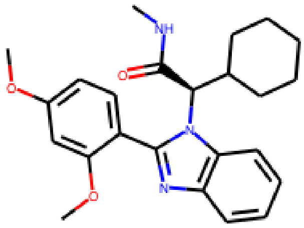

FXR_37

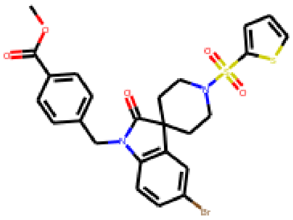

FXR_38

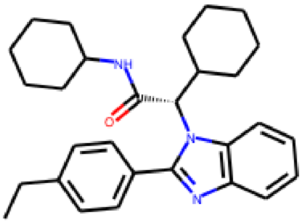

FXR_39

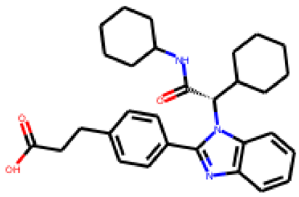

FXR_40

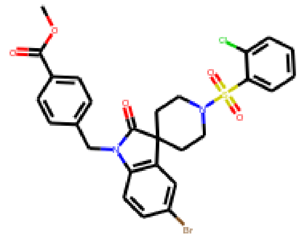

FXR_41

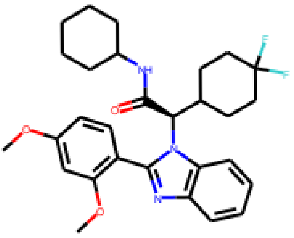

FXR_42

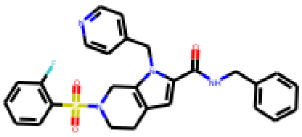

FXR_43

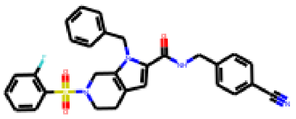

FXR_44

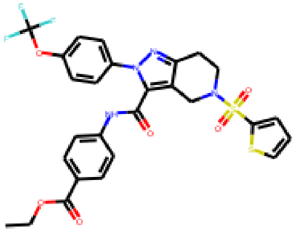

FXR_45

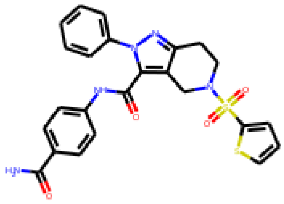

FXR_46

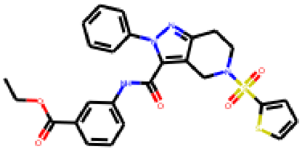

FXR_47

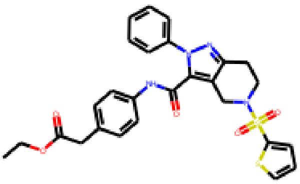

FXR_48

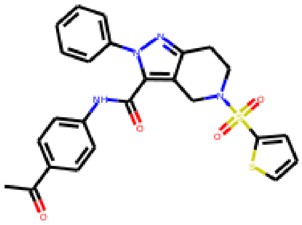

FXR_49

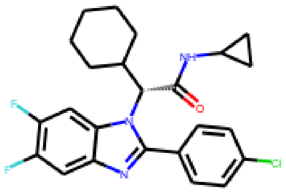

FXR_50

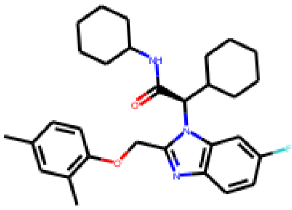

FXR_51

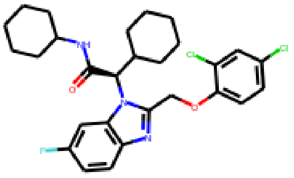

FXR_52

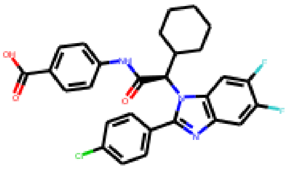

FXR_53

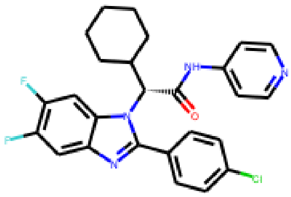

FXR_54

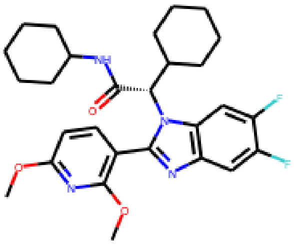

FXR_55

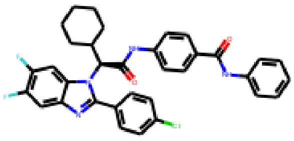

FXR_56

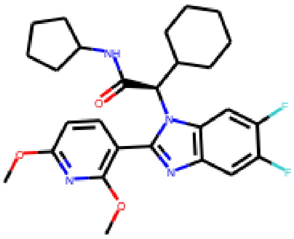

FXR_57

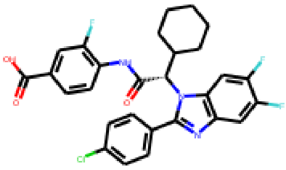

FXR_58

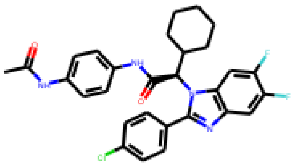

FXR_59

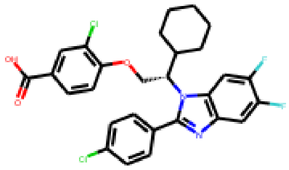

FXR_60

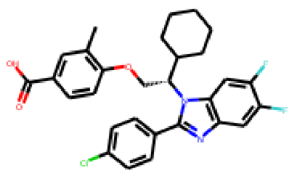

FXR_61

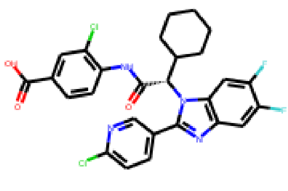

FXR_62

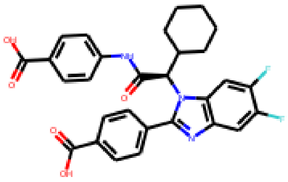

FXR_63

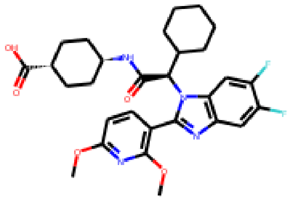

FXR_64

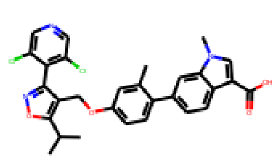

FXR_65

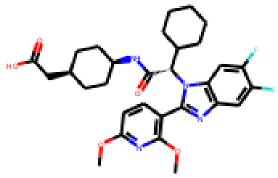

FXR_66

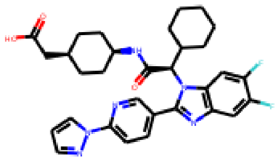

FXR_67

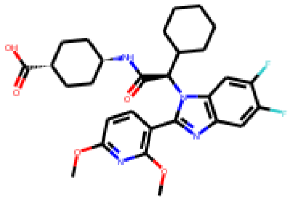

FXR_68

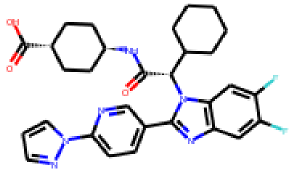

FXR_69

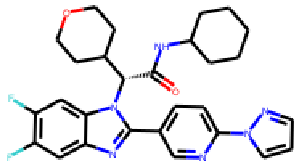

FXR_70

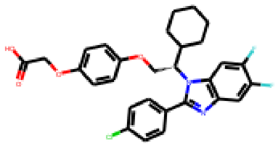

FXR_71

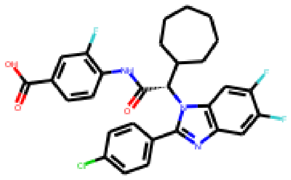

FXR_72

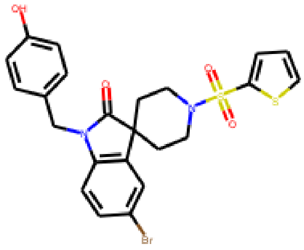

FXR_73

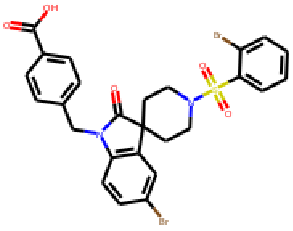

FXR_74

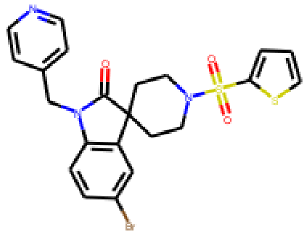

FXR_75

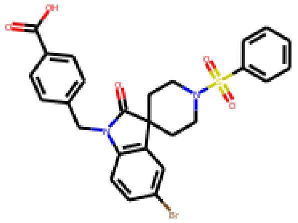

FXR_76

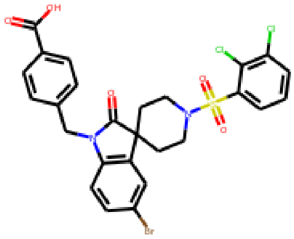

FXR_77

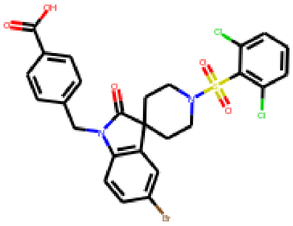

FXR_78

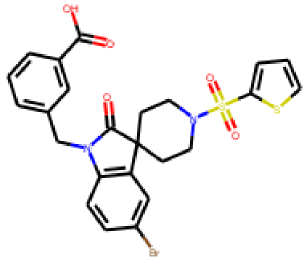

FXR_79

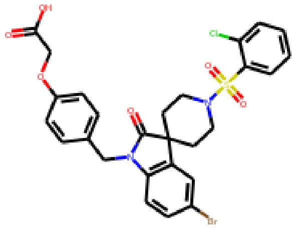

FXR_80

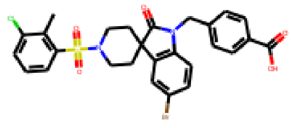

FXR_81

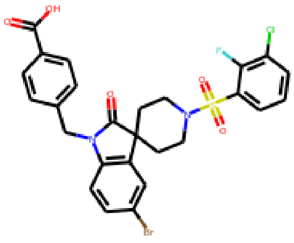

FXR_82

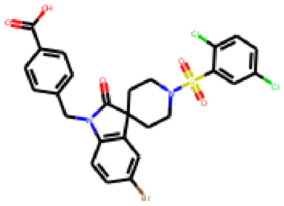

FXR_83

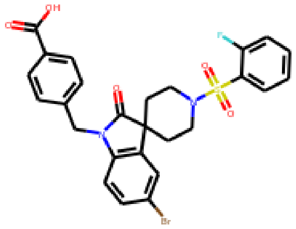

FXR_84

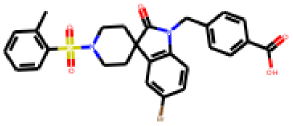

FXR_85

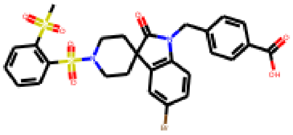

FXR_86

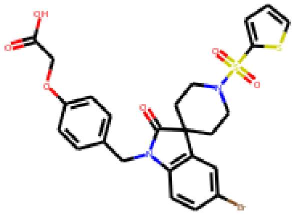

FXR_87

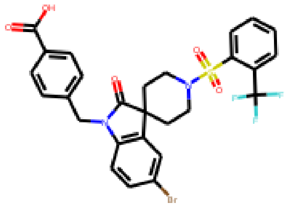

FXR_88

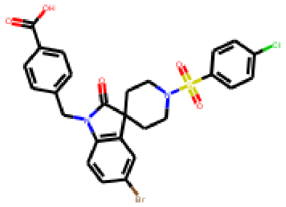

FXR_89

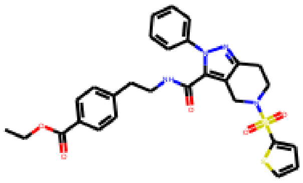

FXR_90

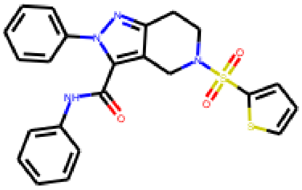

FXR_91

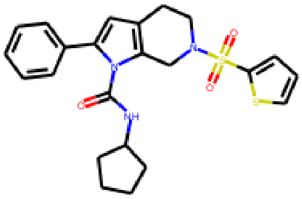

FXR_92

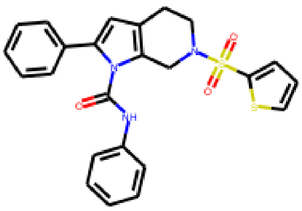

FXR_93

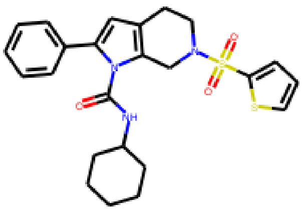

FXR_94

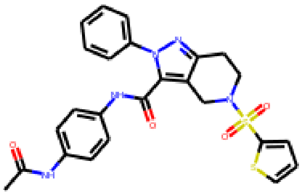

FXR_95

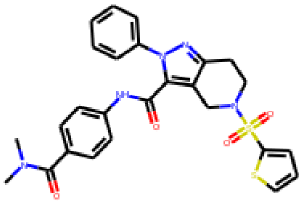

FXR_96

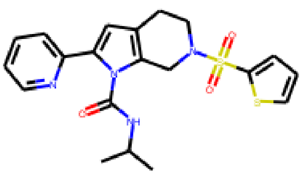

FXR_97

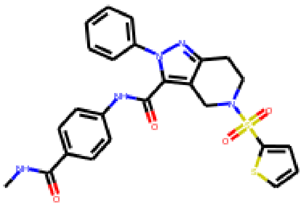

FXR_98

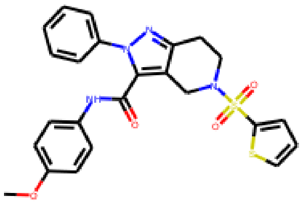

FXR_99

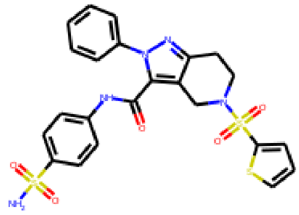

FXR_100

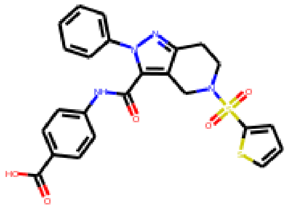

FXR_101

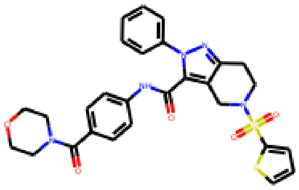

FXR_102

Figure 11: Inhibitor data set consisting of 102 ligands and their 2D structural representations and dataset ID.
